# Supplementary figures and images for: Morphological changes in female reproductive organs in the African monarch butterfly, host to a male-killing Spiroplasma
Source: PeerJ. 2023 Aug 15;11:e15853. doi: 10.7717/peerj.15853 (PMC10437039; doi:10.7717/peerj.15853)

a)

Corpus bursa area (wing size-corrected)

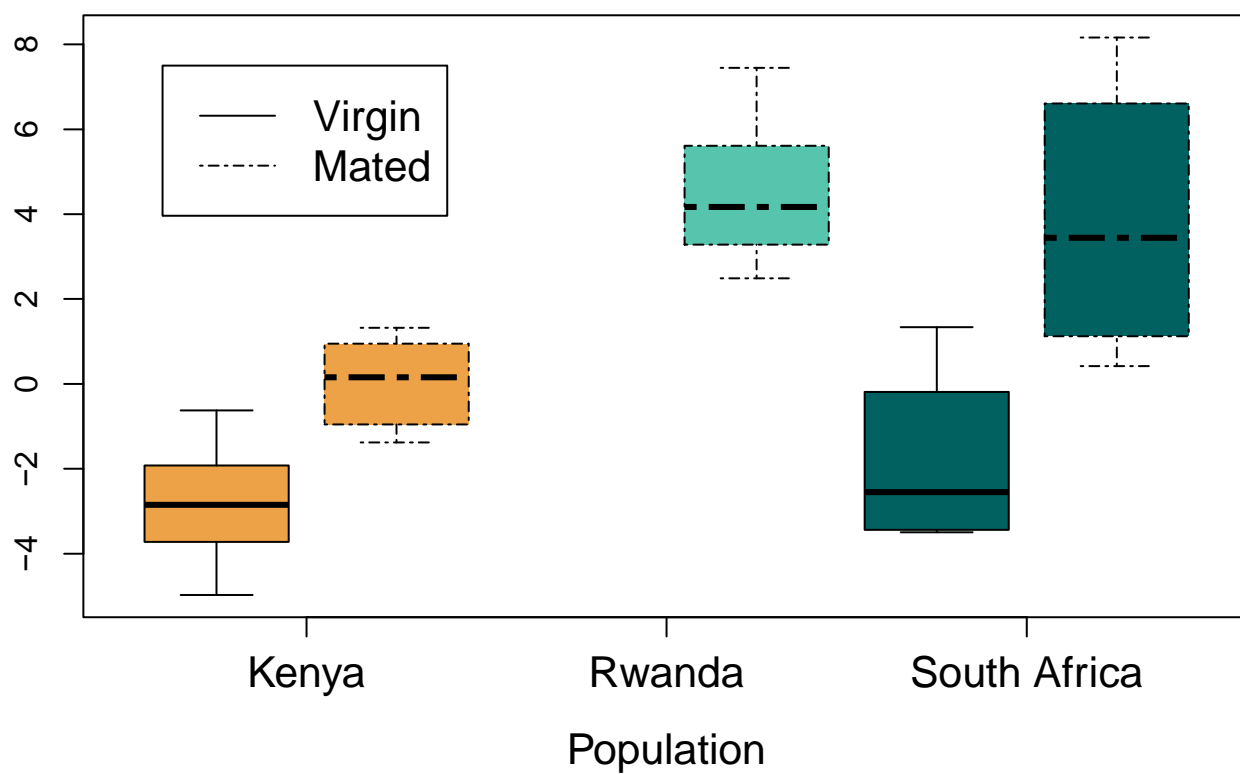

b)

Signum area (wing size-corrected)

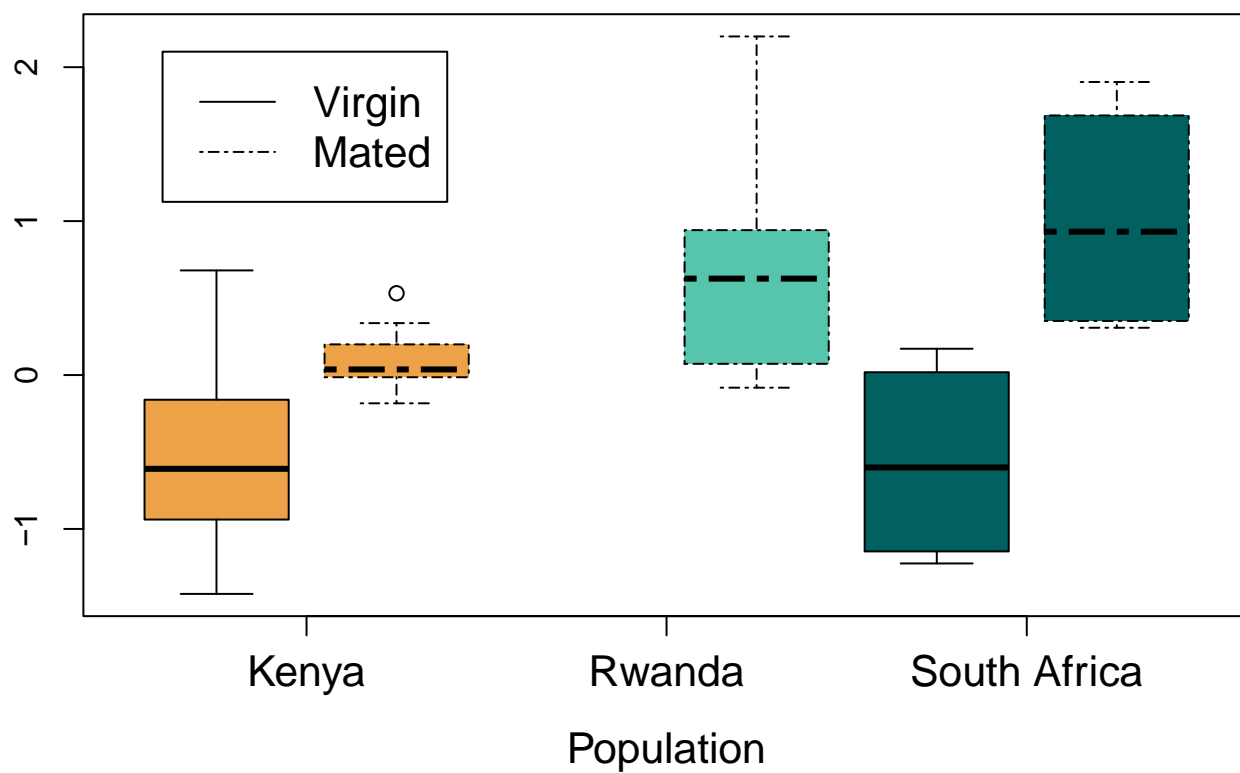

Supplement: Supplemental Information 1 — The boxes represent the interquartile range of the data, and the heavy horizontal lines represent median values. [file peerj-11-15853-s001.pdf]

Wing size (mm)

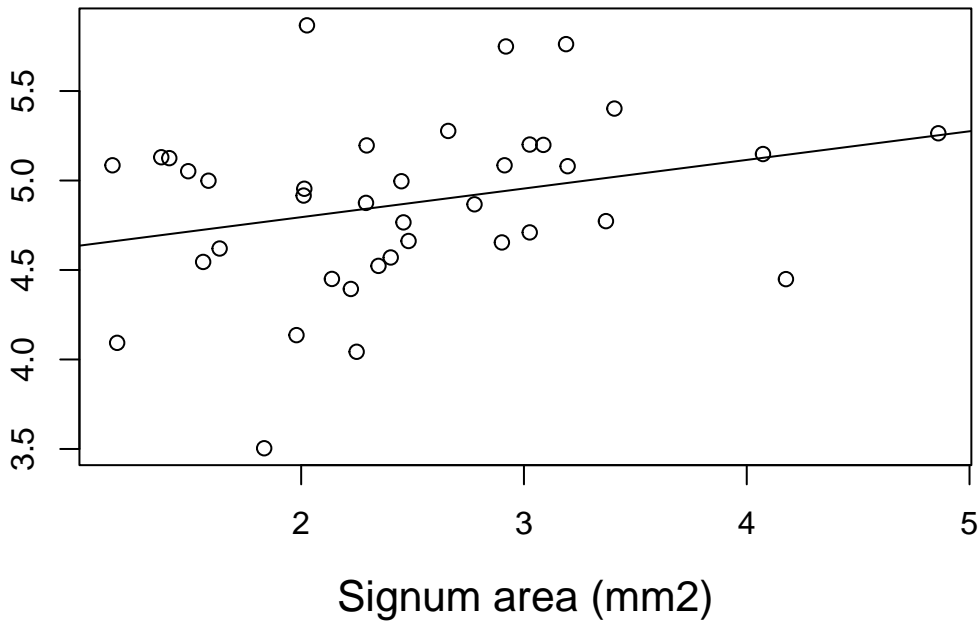

Supplement: Supplemental Information 2 [file peerj-11-15853-s002.pdf]
